# Clinical Feasibility of Fully Sintered (Y, Nb)-TZP for CAD-CAM Single-Unit Restoration: A Pilot Study

**DOI:** 10.3390/ma14112762

**Published:** 2021-05-23

**Authors:** Ki-Won Jeong, Hyung-In Yoon, Jae-Hyun Lee, In-Sung Luke Yeo, Dae-Joon Kim, Jung-Suk Han

**Affiliations:** 1 Department of Prosthodontics, School of Dentistry and Dental Research Institute, Seoul National University, Seoul 03080, Korea; jkw857@gmail.com (K.-W.J.); jhlee.snudh@gmail.com (J.-H.L.); pros53@snu.ac.kr (I.-S.L.Y.); proshan@snu.ac.kr (J.-S.H.); 2 Department of Dentistry, VASIC Research Center, School of Dentistry and Dental Research Institute, Seoul National University, Seoul 03080, Korea; djkim2014@gmail.com

**Keywords:** ceramics, CAD-CAM, zirconia, monolithic, fixed dental prostheses, complication

## Abstract

Fifteen participants (9 male, 6 female) received a total of 15 monolithic single restorations made from fully sintered (Y, Nb)-TZP (tetragonal zirconia polycrystal) block. The restorations were clinically evaluated for survival, success rate, and periodontal probing depths 6 months after the insertion of the restorations. Esthetic, functional, and biological evaluations were also performed over a 6-month follow-up period. The survival and success rates of the single-unit restorations were 100%. The periodontal probing depth values ranged from 1 to 3 mm. No complications with regard to functional and biological properties were observed after 6 months. The postoperative sensitivity was only a transient phenomenon. The fully sintered (Y, Nb)-TZP single-unit restoration showed highly acceptable quality with successful clinical performance over 6 months.

## 1. Introduction

Zirconia is one of the most commonly used ceramics for fixed dental prosthesis (FDP) in dentistry. A 3-mol% yttria-stabilized tetragonal zirconia polycrystal (3Y-TZP) can be fully densified with a fine-grain microstructure, leading to excellent mechanical properties [1]. Currently, with the advancement of computer-aided design and computer-aided manufacturing (CAD-CAM) technology as well as associated restorative materials, it is possible to fabricate an esthetic prosthesis for a single-visit chairside treatment at dental clinics, minimizing extra time and cost, while increasing patient convenience [2]. However, the partially sintered zirconia blocks, which are most widely used for zirconia FDP, are inappropriate for single-visit restorations because these blocks essentially go through the post-milling sintering process for several hours, making it a time-consuming procedure. Furthermore, such blocks should be milled to a size 20–25% larger than the definitive prosthesis to compensate for post-sintering shrinkage, which may cause a poor marginal or internal adaptation of the restoration [3].

As an alternative to the conventional sintering protocol, high-speed sintering for dental zirconia with high temperature in the range of 1570–1590 °C was introduced; this may affect the mechanical properties by increasing the grain size [4,5]. Studies have shown contradictory findings regarding the effect of speed sintering on the mechanical properties, either increasing or decreasing the flexural strength of sintered zirconia [6,7,8,9]. Speed sintering was reported to have a variable influence on the strength, depending on the content of yttria [4,7]. The optical properties of sintered zirconia may also be affected by speed sintering due to an increase in the grain size [4,6,9].

No further heat treatment is required when fabricating zirconia FDP from a fully sintered zirconia block, and it can be milled to its final dimensions without considering the sintering shrinkage [10,11]. The FDP fabricated with fully sintered zirconia has a lower volumetric fraction of pores, higher strength, and more accurate fit than that fabricated with partially sintered zirconia [12,13]. Fully sintered zirconia blocks require robust milling systems with a high level of accuracy due to their high surface hardness, which is twice that of the partially sintered zirconia blocks [10,14]. The milling process of the fully sintered block could increase the wear rate of the milling tool [15,16]. To overcome this issue, an improved fully sintered (Y, Nb)-TZP block with relatively low surface hardness has recently been developed for effective machinability and accuracy [16]. According to the manufacturer, the fully sintered (Y, Nb)-TZP block used in the present study has a lower surface hardness (<9.0 GPa) than fully sintered Y_2_O_3_-stabilized zirconia blocks. In addition, this block is mainly composed of oxygen vacancy deficient zirconia and has a high resistance to low-temperature degradation.

However, there is still a lack of scientific evaluation of fully sintered zirconia prostheses. In particular, no clinical studies involving FDP fabricated using fully sintered (Y, Nb)-TZP blocks are currently available. Thus, the aim of this prospective clinical study was to evaluate the success and survival rates, complications, and prognosis of a single teeth CAD-CAM restoration made of fully sintered zirconia over a 6-month period.

## 2. Materials and Methods

Individuals in need of a single crown restoration were recruited as participants from the Department of Prosthodontics of the University Dental Hospital. The required size of the sample was initially calculated to be 12 using G*power analysis (version 3.1.9.4, Heinrich Heine University Düsseldorf, Düsseldorf, Germany), assuming a probability of Type I error of 0.05, a power of 0.80, a constant proportion of 0.65, and an effect size of 0.30 [17]. In addition, a predicted failure rate of 0.10 and possibility of failure rate in clinical evaluation of 0.10 was assumed. Considering the total failure rate of 0.20, the required number of participants for this study was determined to be 15.

A total of 15 participants requiring a single tooth-borne restoration were recruited based on the following inclusion criteria: (1) successful endodontic treatment, (2) minor tooth fracture limited to the coronal part with no pulpal exposure, and (3) replacement of failed restoration with no pathological condition. Patients in need of post and core treatment due to severe coronal destruction were excluded from this study. Patients with uncontrolled periodontal disease, and heavy smokers (more than 10 cigarettes per day) were also excluded. In addition, each participant voluntarily signed an informed consent form and agreed to be monitored at periodic follow-ups. Between May 2020 and January 2021, each participant was treated by a board-certified prosthodontist or a resident supervised by a board-certified prosthodontist [18].

The shade for each restoration was selected before tooth preparation using the Vitapan classical shade guide (VITA Zahnfabrik, Bad Säckingen, Germany) under corrected light conditions. Tooth preparation was performed based on the clinical guidelines for the anatomically contoured monolithic zirconia restorations [19]. The incisal edge or occlusal surface was reduced by 1.5–2.0 mm, and the facial and lingual axial walls by 1.2–1.5 mm. Line angles were rounded and a chamfer finish with a depth of 0.8–1.2 mm was prepared. The minimum height was set as 4 mm for each abutment tooth with an angle of convergence between 10–12°. After tooth reduction, the full-arch impression was taken with a vinyl polysiloxane impression material with light- and heavy-bodied consistency (Imprint II Garant, 3M, ESPE, MN, USA) using stock metal trays. The provisional restoration was then fabricated with self-curing polymethyl methacrylate (Jet, Lang, IL, USA) and cemented with a eugenol-based temporary cement (Temp bond, Kerr, CA, USA).

**Figure 1:**
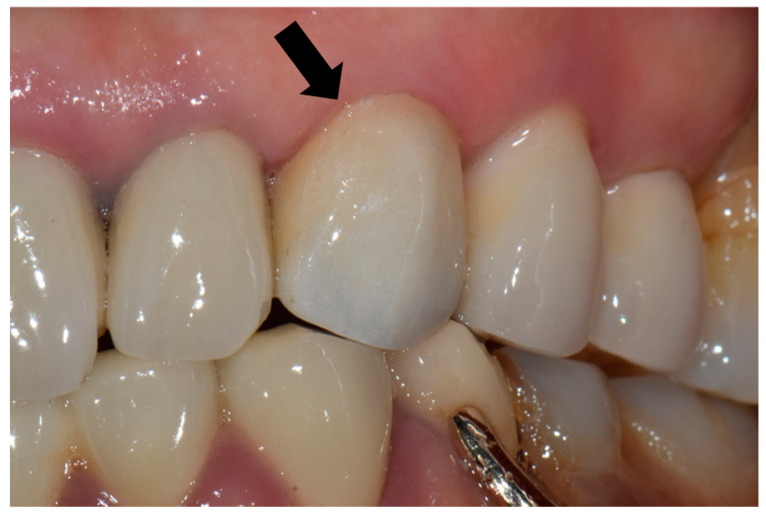
Fully sintered (Y, Nb)-TZP (tetragonal zirconia polycrystal) crown was placed on the maxillary left canine (black arrow).

For each case, the master cast was fabricated with type III dental stone (Snow rock dental stone, DK Mungyo, Gimhae, Korea) and digitally scanned using a model scanner (3Shape D2000, 3Shape, Copenhagen, Denmark). All restorations were designed by dental technicians with 15 years’ experience, using the CAD software (3Shape CAD Design software, 3Shape, Copenhagen, Denmark) following the manufacturer’s instructions. The anatomically contoured zirconia crowns were milled (Cori TEC one, imes-icore, Eiterfeld, Germany) from fully-sintered (Y, Nb)-TZP blocks (Perfit-FS, Vatech MCIS, Gyeonggi-do, South Korea). For optimal shade reproduction, the milled restorations were treated with the zirconia-coloring liquid at 780 °C for 1 min with a firing rate of 45 °C/min using a sintering furnace (Austromat D4, Dekema, Freilassing, Germany). At the third visit, the definitive restoration was carefully evaluated on each participant’s abutment regarding the shape, adaptation, color match, proximal contact, and occlusion (Figure 1). If adjustments were needed, it was conducted using a fine-grained diamond bur with a high-speed handpiece. Resin-modified glass ionomer cement (GC FujiCEM 2, GC, IL, USA) was used to cement the crown in place. The participants were instructed to revisit the clinic for follow-up, as scheduled.

From the fourth visit (r/c-1) to the ninth visit (r/c-6), participants visited once a month, and survival rate, success rate, periodontal probing depth (PPD), and criteria for evaluation of indirect restorations presented by the World Dental Federation (FDI) were examined by each dental clinician in charge [20,21]. The clinicians were calibrated for the evaluation by repeated training on the FDI evaluation criteria from the beginning of the study, supervised by a board-certified prosthodontist with 30-year clinical and research experience (J.-S.H). Survival rate was defined as whether the restoration remained in situ during the follow-up period. Success rate was defined as whether the restoration functioned without complications requiring any intervention. For the gingival health evaluation, the PPD was regularly measured around each abutment tooth separated into six sections (mesio-buccal, mid-buccal, disto-buccal, mesio-lingual, mid-lingual, and disto-lingual). The mean value calculated from the measured PPDs at six different sites was recorded for each participant at each recall. The FDI criteria for evaluation of indirect restorations were divided into three main categories, namely esthetic, functional, and biological properties, which were then subdivided into sub-categories (Table 1). Each sub-category was scored on a scale of 1 to 5, where each number denotes clinically excellent/very good, good, sufficient/satisfactory, unsatisfactory, and poor, in increasing order. In summary, if the quality of the restoration was excellent and fulfilled all the quality criteria, then it would be scored as 1. A score of 2 meant that the quality of the restoration was still highly acceptable, despite one or more deviations from the established criteria. A score of 3 meant that the quality of the restoration was sufficiently acceptable, but with minor shortcomings. A score of 4 meant unacceptable but repairable restorations and a score of 5 required a replacement. The one overall rating of restoration was determined by the highest score among all the sub-category scores.

For the statistical analysis, the measured PPD values were analyzed using the Shapiro–Wilk test for normality. Based on the result of the normality test, the time-dependent statistical comparison of the PPD measurement data was conducted using either the repeated measures analysis of variances (ANOVA) with one within-subject factor (time, six levels) or nonparametric Friedman test. In terms of ANOVA, the Mauchly’s test of sphericity was performed and Greenhouse–Geisser correction was done for the test of within-subjects’ effects if the test result was statistically significant. The significance level of 0.05 was used for the analysis (SPSS 25, SPSS Inc., Chicago, IL, USA).

## 3. Results

Participants included nine male patients with a median age of 60 years and the range of 37 to 80 years, and six female patients with a median age of 63.5 years and the range of 58 to 74 years. Restorations were planned for one incisor, two canines, four premolars, one molar in the maxilla, and seven molars in the mandible (Table 2). All participants attended follow-up visits during the 6-month observation period. After 6 months, the survival and success rates were 100%, and there were no fractures or failures of restorations. The majority of all measured PPDs ranged between 1 and 3 mm (Table 3). Since no statistical significance was found with the Shapiro–Wilk test of normality (*p* > 0.05), the time-dependent comparison of PPD data was performed with the repeated measures ANOVA. The result of Mauchly’s test of sphericity was statistically significant (*p* < 0.001). Based on the repeated measures ANOVA with a Greenhouse–Geisser correction, there was no significant change in the PPD value over time (*p* = 0.390).

According to the FDI criteria, all restorations were considered acceptable in clinical situations. There were no complications in functional properties during the entire follow-up period, while four restorations scored 2 or 3 in esthetic properties and biological properties (Table 4, Table 5, Table 6, Table 7 and Table 8). In esthetic properties, Participant 14 scored 2 in surface staining from the r/c-2 to r/c-6, Participant 8 scored 3 in color stability and translucency from r/c-1 to r/c-6 (Table 7). Regarding biological properties, Participants 2 and 14 scored 2 on r/c-1 and r/c-3, respectively (Table 7). After a 6-month follow-up, in terms of surface staining and color stability, 93.3% of the participants were evaluated as excellent, while 6.7% were as highly or sufficiently acceptable (Table 8). The functional and biologic properties were determined as excellent for 100% of the participants at the end of the observation (Table 8).

## 4. Discussion

In the present study, a total of 15 single CAD-CAM restorations fabricated using fully sintered (Y, Nb)-TZP blocks showed a survival and success rate of 100% over a 6-month follow-up observation. This is in accordance with the previous study, which reported the use of monolithic ceramic as favorable treatment for tooth-supported restoration [22]. Indeed, for such a short observation period, the result is usually likely to be none or only a few unacceptable restorations, so the differentiation of score 1 from 2 becomes more important and the change of these scores can indicate the behavior and the weak points of the restoration.

The most common technical complication during treatment of monolithic zirconia single restorations was the unacceptable color match [23,24]. The final color reproduction of dental zirconia block can be affected by manufacturing process, dental laboratory procedures, and clinical factors [24,25]. In the present study, Participant 8 had a score of 3 for the color stability over the entire observation period. Several factors can affect the color stability of zirconia, such as sintering conditions and surface textures [26,27]. As the fully sintered zirconia block was already sintered under the same conditions, the stainability is closely related to the surface texture, especially the surface roughness [28]. Surface roughness of the yttrium-doped zirconia was important for the longevity of dental restoration, as it had a strong effect on the mechanical strength of the material [29]. In clinical situations, such surface roughness is closely associated with the polishability, which is mainly determined by the surface hardness of the restoration. Park et al. reported that the grain of 3Y-TZP with low surface hardness was easily pulled out by external mechanical damage [30]. As fully sintered (Y, Nb)-TZP exhibits a lower surface hardness than fully sintered 3Y-TZP, it is relatively difficult to obtain a smooth surface by surface polishing. In addition, polished/unpolished ceramics are more stainable than glazed/reglazed ceramics after exposure to coffee [31]. Therefore, when restorations fabricated using fully sintered (Y, Nb)-TZP blocks undergo occlusal adjustment, a more careful polishing procedure is required. Translucency is another important factor responsible for matching the color of natural teeth with restorative materials [24,25]. According to the manufacturer, the fully sintered zirconia used in the present study showed a translucency (42% at 1 mm thickness) comparable with other partially sintered yttria-stabilized zirconia. Therefore, translucency is not an issue in the color matching of fully sintered (Y, Nb)-TZP.

According to the FDI criteria, all restorations exhibited no complications during the entire follow-up visit in terms of functional properties. It implies that fully sintered (Y, Nb)-TZP has sufficient strength and accuracy for single-tooth restoration. Considering the 3-point flexural strength of fully sintered (Y, Nb)-TZP of approximately 700 MPa, as disclosed by the manufacturer, it is capable of enduring a mean occlusal force of approximately 150 N [32]. Fully sintered (Y, Nb)-TZP also exhibited adequate wear resistance since it has relatively higher surface hardness (<9.0 GPa) than glass ceramics [33]. In addition, Janyavula et al. reported that polished monolithic zirconia can provide more acceptable opposing tooth wear than glazed zirconia, veneering porcelain or enamel [34]. For marginal fit accuracy, Cho et al. showed that the trueness outcomes of single restorations fabricated by fully sintered (Y, Nb)-TZP were clinically acceptable [16]. Consequently, carefully polished restorations fabricated using fully sintered (Y, Nb)-TZP can exhibit no fracture, less material and opposing tooth wear, and good marginal adaptation, as shown in the present study. However, the long-term stability of fully sintered zirconia should be investigated.

Postoperative sensitivity and tooth vitality, one of the most common early biological complications, was the only biological complication observed in this study [23]. Roediger et al. mentioned that postoperative sensitivity was a common complication and occurred within the first 13 months of cementation, and it was usually a transient symptom [35]. Likewise, in the present study, postoperative sensitivity appeared in both participants (2 and 14) who scored 2 at first and third follow-up visits, and disappeared by itself at the next visit. Postoperative sensitivity may occur owing to the type of cement, the extent of tooth preparation, inappropriate provisional restorations, removal of the smear layer, presence of occlusal discrepancies, and patient age [36]. According to Pihlaja et al., another very common early biological complication is gingival irritation [23]. In contrast, Worni et al. reported that there was no significant change in the periodontal health of the abutment for a zirconia-based fixed partial denture [37]. In the present study, no periodontal response was found, and the PPD of all participants only varied in the 1 mm range within 1–3 mm. In general, PPD <3 mm was considered to be within the normal range in clinical situations [38]. These results can be attributed to the nature of zirconia itself, which exhibits a lower plaque accumulation than other glass ceramics [39]. However, a 3-year long-term study reported that abutment teeth suffered from increased PPD and periodontal problems [40]. According to Litonjua et al., instructing participants to take adequate home care was more important than the material itself [41]. Therefore, continuous periodontal care is necessary to maintain a healthy periodontal condition.

The limitations of the present study include the fact that the location of the treated tooth varied from participant to participant. As tooth location is associated with varying occlusal force and esthetic sensitivity, it can affect clinical outcomes. Second, the calculated sample size in this study could be relatively small to clearly assess the clinical findings for this 6-month observation. Third, the present study was a short-term study. Although early complications could imply issues with the material properties rather than patient factors [23,24], short-term observation was insufficient to represent the overall properties of the material. Therefore, long-term clinical studies should be conducted to evaluate the properties of the material in detail, as well as patient factors.

## 5. Conclusions

Based on the findings of this prospective clinical trial regarding the properties of the single restoration fabricated by fully sintered zirconia during the 6-month follow-up, the following conclusions were drawn:

1. The survival and success rate of fully sintered (Y, Nb)-TZP single-unit restorations at 6 months were 100%.

2. According to the FDI criteria, the fully sintered (Y, Nb)-TZP single-unit restoration was evaluated as more than sufficiently acceptable in a clinical situation over 6 months.

3. After a 6-month follow-up, no complications with regard to functional and biological properties were observed. The postoperative sensitivity and tooth vitality problem was only a transient phenomenon.

## Figures and Tables

**Table 1:** World dental federation (FDI) criteria for evaluation of indirect restorations.

Esthetic Properties	Functional Properties	Biological Properties
Surface luster	Fractures and retention	Postoperative sensitivity and tooth vitality
Surface staining	Marginal adaptation	Recurrence of caries, erosion, abfraction
Color stability and translucency	Wear	Tooth integrity (enamel cracks)
Anatomic form	Contact point/food impact	Periodontal response (always compared to a reference tooth)
	Radiographic examination (when applicable)	Adjacent mucosa
	Patient’s view	Oral and general health

**Table 2:** Distribution of restorations according to the tooth location. Tooth numbers 1 to 8 sequentially correspond to the teeth in a quadrant (from anterior to posterior).

Tooth	Maxilla	Mandible
Central incisor	1	0
Lateral incisor	0	0
Canine	2	0
1st premolar	4	0
2nd premolar	0	0
1st molar	1	3
2nd molar	0	4
3rd molar	0	0
Total	8	7

**Table 3:** Periodontal probing depth (mm) around a single-unit restoration for each participant during 6-month follow-up.

Follow-Up	Participants														
	#1	#2	#3	#4	#5	#6	#7	#8	#9	#10	#11	#12	#13	#14	#15
r/c-1	1.8	1.8	3.0	2.0	1.7	2.3	1.5	2.3	2.2	2.2	2.0	2.3	3.0	2.3	1.5
r/c-2	1.8	1.8	2.8	2.0	2.8	2.8	1.3	2.0	2.8	2.2	2.0	1.8	3.0	2.5	1.5
r/c-3	1.8	2.0	2.8	2.0	2.8	2.5	1.3	2.0	2.7	2.2	2.0	1.8	3.5	2.5	1.5
r/c-4	1.8	2.0	3.0	2.0	2.8	2.8	1.3	2.0	2.7	2.2	2.0	1.8	3.5	2.5	1.5
r/c-5	1.8	2.0	2.8	2.0	2.8	2.3	1.3	2.0	2.5	2.2	2.0	1.8	3.5	2.5	1.5
r/c-6	1.8	2.0	3.0	2.0	2.5	2.2	1.3	2.0	2.5	2.2	2.0	1.8	3.5	2.5	1.5

**Table 4:** Scores according to the FDI criteria for esthetic properties during 6-month follow-up evaluation.

Follow-Up	Participants														
	#1	#2	#3	#4	#5	#6	#7	#8	#9	#10	#11	#12	#13	#14	#15
r/c-1	1	1	1	1	1	1	1	3	1	1	1	1	1	1	1
r/c-2	1	1	1	1	1	1	1	3	1	1	1	1	1	2	1
r/c-3	1	1	1	1	1	1	1	3	1	1	1	1	1	2	1
r/c-4	1	1	1	1	1	1	1	3	1	1	1	1	1	2	1
r/c-5	1	1	1	1	1	1	1	3	1	1	1	1	1	2	1
r/c-6	1	1	1	1	1	1	1	3	1	1	1	1	1	2	1

**Table 5:** Scores according to the FDI criteria for functional properties during 6-month follow-up evaluation.

Follow-Up	Participants														
	#1	#2	#3	#4	#5	#6	#7	#8	#9	#10	#11	#12	#13	#14	#15
r/c-1	1	1	1	1	1	1	1	1	1	1	1	1	1	1	1
r/c-2	1	1	1	1	1	1	1	1	1	1	1	1	1	1	1
r/c-3	1	1	1	1	1	1	1	1	1	1	1	1	1	1	1
r/c-4	1	1	1	1	1	1	1	1	1	1	1	1	1	1	1
r/c-5	1	1	1	1	1	1	1	1	1	1	1	1	1	1	1
r/c-6	1	1	1	1	1	1	1	1	1	1	1	1	1	1	1

**Table 6:** Scores according to the FDI criteria for biological properties during 6-month follow-up evaluation.

Follow-Up	Participants														
	#1	#2	#3	#4	#5	#6	#7	#8	#9	#10	#11	#12	#13	#14	#15
r/c-1	1	2	1	1	1	1	1	1	1	1	1	1	1	1	1
r/c-2	1	1	1	1	1	1	1	1	1	1	1	1	1	1	1
r/c-3	1	1	1	1	1	1	1	1	1	1	1	1	1	2	1
r/c-4	1	1	1	1	1	1	1	1	1	1	1	1	1	1	1
r/c-5	1	1	1	1	1	1	1	1	1	1	1	1	1	1	1
r/c-6	1	1	1	1	1	1	1	1	1	1	1	1	1	1	1

**Table 7:** Complications of a single crown (n = 15) during 6-month follow-up evaluation.

Main Category	Sub-Category	Participants	Score	Appearance
Esthetic properties	Surface staining	#14	2	r/c-2,3,4,5,6
	Color stability and translucency	#8	3	r/c-1,2,3,4,5,6
Biological properties	Postoperative sensitivity and tooth vitality	#2 #14	2 2	r/c-1 r/c-3

**Table 8:** Distribution of scores according to the FDI criteria for esthetic, functional, and biological properties after 6-month follow-up, in percentage (n = 15).

Category	Score (Scale of 1 to 5)				
	1	2	3	4	5
**Esthetic Properties**					
Surface luster	100 (15)	-	-	-	-
Surface staining	93.3 (14)	6.7 (1)	-	-	-
Color stability and translucency	93.3 (14)	-	6.7 (1)	-	-
Anatomic form	100 (15)	-	-	-	-
**Functional Properties**					
Fractures and retention	100 (15)	-	-	-	-
Marginal adaptation	100 (15)	-	-	-	-
Wear	100 (15)	-	-	-	-
Contact point/food impact	100 (15)	-	-	-	-
Radiographic examination	100 (15)	-	-	-	-
**Biological Properties**					
Postoperative sensitivity and tooth vitality	100 (15) *	-	-	-	-
Recurrence of caries, erosion, abfraction	100 (15)	-	-	-	-
Tooth integrity	100 (15)	-	-	-	-
Periodontal response	100 (15)	-	-	-	-
Adjacent mucosa	100 (15)	-	-	-	-
Oral and general health	100 (15)	-	-	-	-

* For participants 2 and 14, the scores were graded as 2 at r/c-1 and r/c-3, respectively. However, all the scores were graded as 1 at the time of r/c-6.

## Data Availability

The data presented in this study are available on request from the corresponding author.

## References

[1] Piconi C., Maccauro G. (1999). Zirconia as a ceramic biomaterial. Biomaterials.

[2] Sannino G., Germano F., Arcuri L., Bigelli E., Arcuri C., Barlattani A. (2014). Cerec CAD/CAM chairside system. ORAL Implantol..

[3] Rezende C.E.E., Borges A.F.S., Macedo R.M., Rubo J.H., Griggs J.A. (2017). Dimensional changes from the sintering process and fit of Y-TZP copings: Micro-CT analysis. Dent. Mater..

[4] Jansen J.U., Lümkemann N., Letz I., Pfefferle R., Sener B., Stawarczyk B. (2019). Impact of high-speed sintering on translucency, phase content, grain sizes, and flexural strength of 3Y-TZP and 4Y-TZP zirconia materials. J. Prosthet. Dent..

[5] Stawarczyk B., Özcan M., Hallmann L., Ender A., Mehl A., Hämmerlet C.H. (2013). The effect of zirconia sintering temperature on flexural strength, grain size, and contrast ratio. Clin. Oral. Investig..

[6] Cokic S.M., Vleugels J., Van Meerbeek B., Camargo B., Willems E., Li M., Zhang F. (2020). Mechanical properties, aging stability and translucency of speed-sintered zirconia for chairside restorations. Dent. Mater..

[7] Jerman E., Wiedenmann F., Eichberger M., Reichert A., Stawarczyk B. (2020). Effect of high-speed sintering on the flexural strength of hydrothermal and thermo-mechanically aged zirconia materials. Dent. Mater..

[8] Kauling A.E., Güth J.F., Erdelt K., Edelhoff D., Keul C. (2020). Influence of speed sintering on the fit and fracture strength of 3-unit monolithic zirconia fixed partial dentures. J. Prosthet. Dent..

[9] Lawson N.C., Maharishi A. (2020). Strength and translucency of zirconia after high-speed sintering. J. Esthet. Restor. Dent..

[10] Abduo J., Lyons K., Swain M. (2010). Fit of zirconia fixed partial denture: A systematic review. J. Oral. Rehabil..

[11] Sinmazisik G., Demirbas B., Tarcin B. (2014). Influence of dentin and core porcelain thickness on the color of fully sintered zirconia ceramic restorations. J. Prosthet. Dent..

[12] Ahmed W.M., Troczynski T., McCullagh A.P., Wyatt C.C., Carvalho R.M. (2019). The influence of altering sintering protocols on the optical and mechanical properties of zirconia: A review. J. Esthet. Restor. Dent..

[13] Kohorst P., Brinkmann H., Li J., Borchers L., Stiesch M. (2009). Marginal accuracy of four-unit zirconia fixed dental prostheses fabricated using different computer-aided design/computer-aided manufacturing systems. Eur. J. Oral. Sci..

[14] Denry I., Kelly J.R. (2008). State of the art of zirconia for dental applications. Dent. Mater..

[15] Tinscherta J., Nattb G., Hassenpflugb S., Spiekermanna H. (2004). Status of Current CAD/CAM Technology in Dental Medicine Stand der aktuellen CAD/CAM-Technik in der Zahnmedizin. Int. J. Comput. Dent..

[16] Cho J.H., Yoon H.I., Han J.S., Kim D.J. (2019). Trueness of the Inner Surface of Monolithic Crowns Fabricated by Milling of a Fully Sintered (Y, Nb)-TZP Block in Chairside CAD–CAM System for Single-visit Dentistry. Materials.

[17] Faul F., Erdfelder E., Buchner A., Lang A.G. (2009). Statistical power analyses using G* Power 3.1: Tests for correlation and regression analyses. Behav. Res. Methods..

[18] Manhart J., Scheibenbogen-Fuchsbrunner A., Chen H.Y., Hickel R. (2000). A 2-year clinical study of composite and ceramic inlays. Clin. Oral. Investig..

[19] Bachhav V.C., Aras M.A. (2011). Zirconia-based fixed partial dentures: A clinical review. Quintessence Int..

[20] Hickel R., Roulet J.F., Bayne S., Heintze S.D., Mjör I.A., Peters M., Rousson V., Randall R., Schmalz G., Tyas M. (2007). Recommendations for conducting controlled clinical studies of dental restorative materials. Clin. Oral. Investig..

[21] Hickel R., Roulet J.F., Bayne S., Heintze S.D., Mjör I.A., Peters M., Rousson V., Randall R., Schmalz G., Tyas M. (2007). Recommendations for conducting controlled clinical studies of dental restorative materials. Science Committee Project 2/98--FDI World Dental Federation study design (Part I) and criteria for evaluation (Part II) of direct and indirect restorations including onlays and partial crowns. J. Adhes. Dent..

[22] Mazza L.C., Lemos C.A.A., Pesqueira A.A., Pellizzer E.P. (2021). Survival and complications of monolithic ceramic for tooth-supported fixed dental prostheses: A systematic review and meta-analysis. J. Prosthet. Dent..

[23] Pihlaja J., Näpänkangas R., Raustia A. (2014). Early complications and short-term failures of zirconia single crowns and partial fixed dental prostheses. J. Prosthet. Dent..

[24] Lestan N.G., Özcan M., Kocjan A., Oblak Č. (2021). Clinical evaluation of monolithic zirconia multiunit posterior fixed dental prostheses. J. Prosthet. Dent..

[25] Tabatabaian F. (2019). Color aspect of monolithic zirconia restorations: A review of the literature. J. Prosthodont..

[26] Ebeid K., Wille S., Hamdy A., Salah T., El-Etreby A., Kern M. (2014). Effect of changes in sintering parameters on monolithic translucent zirconia. Dent. Mater..

[27] Sulaiman T.A., Abdulmajeed A.A., Donovan T.E., Vallittu P.K., Närhi T.O., Lassila L.V. (2015). The effect of staining and vacuum sintering on optical and mechanical properties of partially and fully stabilized monolithic zirconia. Dent. Mater. J..

[28] Kursoglu P., Motro P.F.K., Kazazoglu E. (2014). Correlation of surface texture with the stainability of ceramics. J. Prosthet. Dent..

[29] Marrelli M., Maletta C., Inchingolo F., Alfano M., Tatullo M. (2013). Three-point bending tests of zirconia core/veneer ceramics for dental restorations. Int. J. Dent..

[30] Park C., Vang M.S., Park S.W., Lim H.P. (2017). Effect of various polishing systems on the surface roughness and phase transformation of zirconia and the durability of the polishing systems. J. Prosthet. Dent..

[31] Motro P.F.K., Kursoglu P., Kazazoglu E. (2012). Effects of different surface treatments on stainability of ceramics. J. Prosthet. Dent..

[32] Kohyama K., Hatakeyama E., Sasaki T., Dan H., Azuma T., Karita K. (2004). Effects of sample hardness on human chewing force: A model study using silicone rubber. Arch. Oral. Biol..

[33] Zhang F., Spies B.C., Vleugels J., Reveron H., Wesemann C., Müller W.D., van Meerbeek B., Chevalier J. (2019). High-translucent yttria-stabilized zirconia ceramics are wear-resistant and antagonist-friendly. Dent. Mater..

[34] Janyavula S., Lawson N., Cakir D., Beck P., Ramp L.C., Burgess J.O. (2013). The wear of polished and glazed zirconia against enamel. J. Prosthet. Dent..

[35] Roediger M., Gersdorff N., Huels A., Rinke S. (2010). Prospective evaluation of zirconia posterior fixed partial dentures: Four-year clinical results. Int. J. Prosthodont..

[36] Hilton T., Hilton D., Randall R., Ferracane J.L. (2004). A clinical comparison of two cements for levels of post-operative sensitivity in a practice-based setting. Oper. Dent..

[37] Worni A., Katsoulis J., Kolgeci L., Worni M., Mericske-Stern R. (2017). Monolithic zirconia reconstructions supported by teeth and implants: 1-to 3-year results of a case series. Quintessence Int..

[38] Greenstein G. (1997). Contemporary interpretation of probing depth assessments: Diagnostic and therapeutic implications. A literature review. J. Periodontol..

[39] Bremer F., Grade S., Kohorst P., Stiesch M. (2011). In vivo biofilm formation on different dental ceramics. Quintessence Int..

[40] Tartaglia G.M., Sidoti E., Sforza C. (2011). A 3-year follow-up study of all-ceramic single and multiple crowns performed in a private practice: A prospective case series. Clinics.

[41] Litonjua L.A., Cabanilla L.L., Abbott L.J. (2012). Plaque formation and marginal gingivitis associated with restorative materials. Compend. Contin. Educ. Dent..

